# Improvement in the intrinsic water use efficiency of sugarcane by intergeneric hybridization with *Erianthus arundinaceus*

**DOI:** 10.3389/fpls.2025.1649112

**Published:** 2025-11-21

**Authors:** Hiroo Takaragawa, Yoshifumi Terajima, Ken Okamoto

**Affiliations:** Tropical Agriculture Research Front, Japan International Research Center for Agricultural Sciences, Ishigaki, Okinawa, Japan

**Keywords:** assimilation rate, A *vs* g_s_ curve, drought tolerance, intergeneric F_1_ hybrid, photosynthetic rate, stomatal conductance, stomatal density, transpiration efficiency

## Abstract

**Introduction:**

Sugarcane (*Saccharum* spp.) is often grown under unstable rainfall and drought conditions, highlighting the need for improved drought tolerance. *Erianthus arundinaceus*, a closely related species, shows high intrinsic water use efficiency (*iWUE*) and robust root formation capacity. However, research on improving sugarcane leaf traits using *Erianthus* is limited. This study aimed to evaluate the water use efficiency and associated leaf traits of sugarcane × *Erianthus* intergeneric F_1_ hybrids and their parental genotypes under both wet and dry pot conditions in a greenhouse to assess the potential for improving drought tolerance through intergeneric hybridization.

**Methods:**

The sugarcane cultivars (drought-susceptible NiF8 and drought-tolerant Ni9), *Erianthus* accessions (JIRCAS1 and JW630), and their intergeneric F_1_ hybrids (NiF8 × JIRCAS1 and NiF8 × JW630) were evaluated for gas exchange and leaf morphology.

**Results:**

*Erianthus* accessions had superior stomatal responses, lower stomatal conductance, and higher *iWUE* than NiF8, with JW630 showing higher *iWUE* than Ni9. However, *Erianthus* accessions had lower gravimetric water use efficiencies (*gWUE*) than the sugarcane cultivars, likely due to the higher leaf area ratio (LAR). The hybrids displayed higher *iWUE*, with dry matter partitioning characteristics resembling those of sugarcane (low LAR, high shoot/root ratio, and high partitioning to the stem), suggesting potential for higher *gWUE* under field canopy conditions. The high *iWUE*s of *Erianthus* and F_1_ hybrids were suggested to be attributed to the fewer stomata on the abaxial surface.

**Conclusion:**

This study highlights *Erianthus*’s potential in improving leaf characteristics to enhance sugarcane drought tolerance because the hybrids demonstrated “best of both worlds” scenario, where they inherited high *iWUE* from *Erianthus* with favorable biomass partitioning characteristics from sugarcane.

## 1 Introduction

Water deficits or droughts are primary climatic factors that constraint global sugarcane (*Saccharum* spp.) production, regardless of whether the final product is sugar or biomass. Drought stress impairs key physiological functions, including photosynthesis and associated enzymatic activities (Du et al., 1996; Dinh et al., 2017; Marchiori et al., 2017; Zhao et al., 2017), leading to reduced biomass production and lower final yield in sugarcane (Robertson et al., 1999; Basnayake et al., 2012, 2015). Therefore, enhancing water use efficiency (WUE)—crop productivity per amount of water resources applied to the field or used by the plant—is crucial for optimizing the yield and profitability of sugarcane production under both rainfed and irrigated conditions (Basnayake et al., 2012; Natarajan et al., 2020), which also could save water resource. Yield stability under variable water conditions can be achieved through improved breeding strategies and effective crop management (Ferreira et al., 2017; Singels et al., 2019; Dlamini, 2021; Watanabe et al., 2021). However, despite advanced crop management techniques, variety selection remains essential, as varieties are typically classified as either drought-tolerant or drought-susceptible (Inman-Bamber and Stead, 1990). While the development of new drought-tolerant varieties through breeding is considered the most effective strategy for offering growers viable options, the limited availability of such varieties in drought-prone regions and cropping seasons suggests that breeding and selection efforts are currently insufficient in terms of efficiency (Acreche, 2017). This is likely due to the difficulty in evaluating the impact of environmental factors on WUE, as the sugarcane growth period is long and the effects of these factors are substantial and complex (Basnayake et al., 2012; Liu et al., 2016; Natarajan et al., 2020). Therefore, scaling down WUE along both the time/phenology axis and the size axis is considered effective to help understand the physiological mechanisms of WUE. Intrinsic WUE (*iWUE*), also known as transpiration efficiency, which is the ratio of individual leaf photosynthetic rate (*A*) to its transpiration indicator, namely stomatal conductance (*g_s_*), is recognized as the minimum unit of WUE and has been proposed as an important target for crop breeding (Condon et al., 2004). Besides a ratio of *A* and *g_s_*, stomatal responsiveness, indicated by *A vs*. *g_s_* regression, is also important to consider determinants for WUE (Battle et al., 2024).

The narrow genetic base of previous sugarcane cultivars, with several specific genotypes in their pedigrees, is a major limitation for enhancing yield and stress tolerance (Wei and & Jackson, 2016; Jackson, 2019). *Erianthus*, a genus within the closely related *Saccharum* complex, is considered a promising genetic resource for sugarcane improvement, having played a key role in the establishment of sugarcane species (Jackson and Henry, 2011). Among species in *Saccharum* complex such as *S.* sp*ontaneum* and *Miscanthus*, *Erianthus* has been reported to exhibit exceptional tolerance to wider range of biotic and abiotic stresses, including nematodes (Bhuiyan et al., 2014, 2016), drought (Matsuo et al., 2002; Augustine et al., 2015; Manoj et al., 2019), soil acidity (Matsuo et al., 2002; Takaragawa et al., 2023), and salt (Manoj et al., 2019). Although limited agronomic studies have focused on its vigorous growth and stress tolerance (Jackson and Henry, 2011), the morphological and physiological traits contributing to its resilience are becoming increasingly understood. The robust growth of *Erianthus* is often linked to its high root-forming capacity (Matsuo et al., 2002; Takaragawa et al., 2022; Terajima et al., 2023). However, both above-ground and below-ground characteristics contribute to its drought tolerance. For instance, *Erianthus* exhibits higher *iWUE*, as indicated by the gas exchange properties of individual leaves under well-watered conditions, compared to commercial sugarcane cultivars, even in a limited root zone under pot culture (Jackson et al., 2016; Li et al., 2017). Furthermore, *Erianthus* showed higher *iWUE* than sugarcane when grown in pots under both wet and dry soil conditions, attributed to factors such as lower stomatal density on the abaxial surface of the leaves and the accumulation of specific leaf metabolites, including betaine and GABA (Takaragawa and Wakayama, 2024). *Erianthus* has been used for intergeneric hybridization to improve sugarcane productivity traits, with reported gains in biomass productivity (Nair et al., 2017; Pachakkil et al., 2019; Meena et al., 2020) and root system characteristics (Fukuhara et al., 2013; Bhuiyan et al., 2016; Takaragawa et al., 2022; Terajima et al., 2023). However, reports on the improvement of sugarcane leaf traits using *Erianthus* are limited.

Closely related genetic resources other than *Erianthus* have been used to improve gas exchange characteristics and stomatal morphology in sugarcane. Hybridization with *S. officinarum* and *S.* sp*ontaneum* improved leaf morphology, including stomatal distribution and leaf width, and photosynthetic characteristics of the interspecific hybrid F_1_ (Rao, 1951; Irvine, 1975). Additionally, interspecific hybridization between commercial sugarcane cultivars and *S.* sp*ontaneum* has led to improvement in leaf anatomical characteristics such as leaf thickness and cellular arrangement (Jumkudling et al., 2022). Furthermore, intergeneric hybrids between sugarcane cultivars and *Miscanthus* germplasm have demonstrated improved photosynthetic capacity at low temperatures (Glowacka et al., 2016; Kar et al., 2019, 2020). Investigation of leaf traits related to drought tolerance in *Erianthus* using back cross (BC)_1_F_1_ lines of *S. officinarum* and *E. arundinaceus* suggested potential improvements in metabolites such as proline and several enzymes through intergeneric hybridization (Deng et al., 2019). However, the benefits of intergeneric hybridizations could not be demonstrated due to the low composition of *Erianthus*-derived genes owing to the extensive backcrossing to sugarcane. Moreover, the authors compared the hybrid lines with major sugarcane cultivars rather than with the parental genotypes of sugarcane and *Erianthus*. The gas exchange characteristics of sugarcane cultivars and interspecific/intergeneric hybrids have been compared, but without using parental genotypes as reference controls (Jackson et al., 2016; Li et al., 2017). Therefore, a more comprehensive evaluation of the intergeneric hybrid F_1_, including both parental genotypes as comparators, is necessary to assess the potential for introducing the superior leaf traits of *Erianthus* into sugarcane. Additionally, no studies have examined the response of leaf characteristics such as physiological and anatomical traits under soil drying conditions in intergeneric hybrids with *Erianthus*.

Therefore, in this study, we aimed to investigate the water use efficiency and associated leaf characteristics of sugarcane × *Erianthus* intergeneric F_1_ hybrids and their parental genotypes under both wet and dry pot conditions in a greenhouse to assess the potential for improving drought tolerance through intergeneric hybridization.

## 2 Materials and methods

### 2.1 Plant materials and treatments

The sugarcane cultivars NiF8 (drought-susceptible) and Ni9 (drought-tolerant); *Erianthus* accessions JIRCAS1 (unknown origin) and JW630 (collected in Shizuoka, Japan); and their intergeneric hybrids F_1_ J08-12 (NiF8 × JIRCAS1) and J16-77 (NiF8 × JW630) were included in the study. The hybrids J08–12 and J16–77 were confirmed true intergeneric hybrids using PCR-based simple sequence repeat (SSR) markers (D'Hont et al., 1995) and nuclear DNA content by flow cytometry assays (Pachakkil et al., 2019) (**Supplementary Figure S1**). Two *Erianthus* accessions are known to belong to genetically distinct groups (Tsuruta et al., 2012, 2017) and both show robust root system in the field (Terajima et al., 2023). The plants were grown in a temperature- and humidity-controlled glasshouse at the Tropical Agriculture Research Front, Japan International Research Center for Agricultural Sciences (24°22'43" N, 124°11'4" E). The day temperature was maintained at 31°C from 7:00 am to 7:00 pm, while the night temperature was set at 27°C; the relative humidity was maintained at 60% (**Supplementary Figure S2**). The daily cumulative solar radiation in the greenhouse during the growing season averaged 12.4 ± 5.1 mol m^-2^ day^-1^.

The pot experiment was performed with a two-factorial design examining sugarcane genotypes and soil moisture conditions (6 genotypes × 2 water regimes). Seedlings of single-bud setts were raised in containers filled with potting mix (Minori, JA Okinawa, Okinawa, Japan) from May 14 (for *Erianthus*) and June 2 (for sugarcane and intergeneric hybrids) 2021. Due to the slower germination and initial growth of *Erianthus*, their seedlings were germinated approximately two weeks earlier to synchronize with the growth stage of the other plants. On July 19, 2021, 12 plants of each genotype were transplanted into 1/2000a Wagner pots filled with 10 kg of FW potting mix. Fertilization was performed at transplantation using a solid slow-release fertilizer with a nutrient ratio of N:P:K = 2.4:0.7:1.0 g pot^-1^. To reduce evaporation, cobble gravel was spread at a depth of 2 cm on the soil surface, as described in Jackson et al. (2016). Irrigation was initially provided three times daily using an automatic drip system until irrigation control began. On August 18, 2021, drainage was stopped using rubber plugs, and manual irrigation control was implemented. Irrigation was controlled according to Dinh et al. (2017) by reading the volumetric water content (VWC) at 8:00 am using a soil moisture sensor (EC-5, Meter) placed at a soil depth of 13 cm (center of the pot) and estimating the water consumption per pot from the previously obtained soil bulk density. Beginning August 25, 2021, the soil pF value was estimated from the VWC (**Supplementary Figure S4**) using the moisture characteristic curve of the test soil obtained earlier (**Supplementary Figure S3**). Two treatments were applied: a wet treatment where the soil was irrigated to a well-watered condition (0.445 m^3^ m^-3^; pF 1.4), and a dry treatment, where irrigation was gradually reduced by approximately 1% until reaching the permanent wilt point (0.131 m^3^ m^-3^; pF 4.2). The pots were randomly placed with four replicates per treatment.

### 2.2 Measurement of gas exchange parameters

The gas exchange parameters—photosynthetic rate (*A*), stomatal conductance (*g_s_*), transpiration rate (*E*), and intercellular CO_2_ concentration (*C_i_*)—of the uppermost fully expanded leaves were measured using a portable gas exchange measurement device (LI-6400, LI-COR BioSciences, Lincoln, Nebraska, USA) on August 24 (prior to the start of the irrigation treatment), and on September 6, 16, 23, and October 1 during the treatment period in 2021. A 6-cm^2^ (2 cm × 3 cm) LED chamber (LI-6400B, LI-COR) was used, with two light intensity levels: unsaturated (500 µmol m^-2^ s^-1^) and saturated (2000 µmol m^-2^ s^-1^) photosynthetic photon flux density (PPFD). Light curves previously measured for NiF8 and JW630 confirmed no difference between the two species regarding light saturation and unsaturation (Takaragawa and Matsuda, 2023). The flow rate and reference CO_2_ concentration were set to 400 µmol s^-1^ and 400 µmol mol^-1^, respectively. Leaf temperature was maintained at 30.9 ± 1.0°C via a block temperature set at 30°C. Leaf vapor pressure deficit (VPD) was manually controlled at 1.9 ± 0.2 kPa using a desiccant bulb filled with Drierite® (W. A. Hammond Drierite Co., Xenia, OH, USA). *iWUE* was calculated from the obtained *A* and *g_s_*, using the equation:


$$iWUE\;\left(\text{µmol mo}{\text{l}}^{-1}\right)=A/g_{\text{s}}$$

The choice of *g_s_* to calculate gas exchange water use efficiency is based on its role as a transpiration index that accounts for VPD. This approach is easier and equitable, facilitating comparison across studies. In contrast to using transpiration rate or photosynthetic water use efficiency (*A*/*E*), *g_s_* provides a more consistent and fairer metric for comparison with other literatures (Jackson et al., 2016; Li et al., 2017; Nakabaru et al., 2020).

### 2.3 Survey of leaf anatomical features

A thin layer of nail polish was applied to both sides of the leaf used for gas exchange measurements, and stomatal samples were collected using double-sided tape (Wu and Zhao, 2017; Takaragawa and Wakayama, 2024). Cross-sections of the tested leaves were prepared manually and fixed onto glass slides to measure the interveinal distance—defined as the distance between vascular bundles. Observations were made using an optical microscope system (Eclipse E800, Nikon, Tokyo, Japan) equipped with image analysis software (NIS-elements, Nikon).

### 2.4 Evaluation of plant growth

At the beginning of the treatment, four plants per genotype were harvested, with all remaining plants harvested 49 days after treatment (on October 13, 2021). The culm length of the main stem, total leaf area, and dry matter weight of each organ were recorded. Leaf area was measured using a leaf area meter (LI-3100, LI-COR). The rate of main-stem elongation during the treatment period was calculated based on the culm length before and after treatment. Underground parts were washed to remove soil and separated into roots and underground stems (stubbles). The underground stem weight was included in the aboveground weight. The leaf area ratio (LAR) during the treatment period was calculated using the leaf area (L_1_, L_2_) and total dry matter weight (W_1_, W_2_) measurements taken before and after the treatment, according to the following equation:


$$\text{LAR }\left(\text{c}{\text{m}}^{2}\text{gD}{\text{W}}^{-1}\right)\;=\frac{\text{ln}{\text{W}}_{2}-\text{ln}{\text{W}}_{1}}{{\text{W}}_{2}-{\text{W}}_{1}}\;\times \frac{{\text{L}}_{2}-{\text{L}}_{1}}{\text{ln}{\text{L}}_{2}-\text{ln}{\text{L}}_{1}}$$

The water use efficiency of biomass production, defined as gravimetric WUE (*gWUE*), was calculated by dividing the increment in dry matter (ΔDW) by the water consumed (ΔWU) during the treatment period, using the following equation (Dinh et al., 2017):


$$gWUE\left(\text{g }{\text{L}}^{-1}\right)=\text{ΔDW}/\text{ΔWU}$$

Total nitrogen content of each plant part was analyzed using an NC analyzer (NC22F; Sumika Chemical Analysis Service, Ltd., Osaka, Japan) to calculate the nitrogen uptake (ΔNU) during the treatment period, and the nitrogen use efficiency (NUE) was calculated using the following equation:


$$NUE\;\left(\text{g g}{\text{N}}^{-1}\right)=\text{ΔDW}/\text{ΔNU}$$

### 2.5 Statistical analysis

Data analysis was conducted using the Bell Curve for Excel statistical analysis software (Social Survey Research Information Co., Ltd., Tokyo, Japan). A two-way factorial analysis of variance (ANOVA) was performed to assess the effects of genotype (six genotypes), water regime (two water regimes), and their interactions on leaf anatomical and dry matter parameters. A four-way factorial ANOVA was also conducted to evaluate the effects of genotype, water regime, PPFD for measurement (two levels), measurement date (five dates), and their interactions with gas exchange parameters. Results of ANOVA were shown with percentage of each factorial variance to total variance. Differences among mean values of the examined parameters for each genotype were determined using Tukey’s test, with statistical significance assumed at *P* < 0.05 (n = 4). Measured *A* and *g_s_* values were plotted for each genotype under each PPFD condition, and a correlation analysis was conducted to derive the *A vs*. *g_s_* slope. Differences in the *A vs*. *g_s_* slope values between NiF8 and each genotype were assessed using a *t*-test, with statistical significance assumed at *P* < 0.05, 0.01, and 0.001.

## 3 Results

### 3.1 Comparison of stomatal responses to drought among genotypes

**Figure 1:**
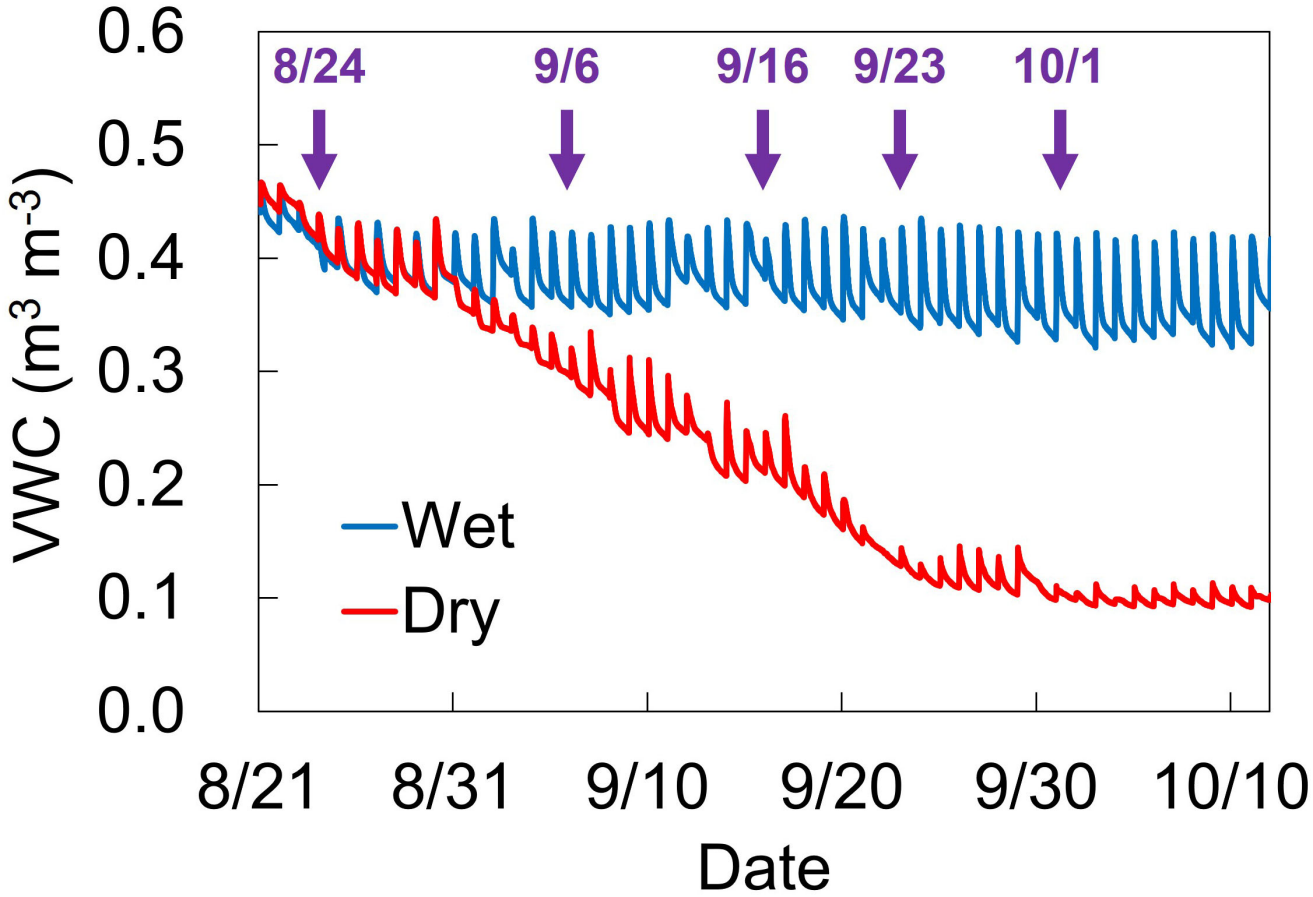
Changes of soil volume water content (VWC) at 15-cm depth under wet and dry conditions. Blue and red lines indicate VWC values under wet and dry conditions, respectively. Manual irrigation control and stress treatment were started from 8/20 and 8/30, respectively. Arrows indicate five dates for gas exchange measurement.

The soil water conditions during the water treatment are shown in **Figure 1**. Based on changes in VWC under dry conditions, leaf gas exchange measurements were performed on August 24, September 6, September 16, September 23, and October 1, with mean VWC values of 0.43, 0.32, 0.25, 0.16, and 0.14 m^3^ m^-3^, respectively (**Table 1**).

**Table 1:** Intrinsic water use efficiency (*iWUE*) of sugarcane, *Erianthus*, and intergeneric F_1_ hybrid under wet and dry conditions.

Date	Genotype		Wet		Dry		Average VWC (%)
			PPFD 500	PPFD 2000	PPFD 500	PPFD 2000	
8/24	Sugarcane	NiF8	72 a	111 ab	62 a	103 a	43.2
		Ni9	93 ab	122 ab	92 abc	127 ab	
	*Erianthus*	JIRCAS1	74 a	105 a	71 ab	108 a	
		JW630	118 b	125 ab	102 bc	120 ab	
	F_1_ hybrid	J08-12 (NiF8 x JIRCAS1)	93 ab	121 ab	93 abc	117 ab	
		J16-77 (NiF8 x JW630)	118 b	136 b	115 c	135 b	
9/6	Sugarcane	NiF8	63 a	105 a	61 a	106 a	31.9
		Ni9	78 ab	120 ab	86 ab	127 a	
	*Erianthus*	JIRCAS1	79 ab	110 ab	71 ab	117 a	
		JW630	91 ab	134 b	120 c	135 a	
	F_1_ hybrid	J08-12 (NiF8 x JIRCAS1)	84 ab	114 ab	98 bc	117 a	
		J16-77 (NiF8 x JW630)	100 b	134 b	96 bc	125 a	
9/16	Sugarcane	NiF8	59 a	98 a	70 a	112 a	24.5
		Ni9	74 ab	113 ab	89 ab	116 a	
	*Erianthus*	JIRCAS1	82 ab	100 a	98 ab	119 a	
		JW630	94 b	114 ab	119 b	125 a	
	F_1_ hybrid	J08-12 (NiF8 x JIRCAS1)	86 ab	117 ab	96 ab	112 a	
		J16-77 (NiF8 x JW630)	94 b	123 b	103 b	122 a	
9/23	Sugarcane	NiF8	53 a	93 a	84 a	128 a	15.5
		Ni9	76 ab	108 bc	119 ab	143 a	
	*Erianthus*	JIRCAS1	80 bc	100 ab	142 b	131 a	
		JW630	83 bc	123 c	137 b	150 a	
	F_1_ hybrid	J08-12 (NiF8 x JIRCAS1)	85 bc	111 bc	110 ab	132 a	
		J16-77 (NiF8 x JW630)	105 c	121 c	145 b	146 a	
10/1	Sugarcane	NiF8	63 a	100 a	133 a	148 a	13.5
		Ni9	84 ab	111 ab	163 ab	154 a	
	*Erianthus*	JIRCAS1	86 ab	101 a	178 b	162 a	
		JW630	93 ab	123 b	164 ab	162 a	
	F_1_ hybrid	J08-12 (NiF8 x JIRCAS1)	87 ab	110 ab	147 ab	147 a	
		J16-77 (NiF8 x JW630)	106 b	122 b	159 ab	161 a	

Different alphabet indicates significant difference between genotypes under each soil water and PPFD conditions at each measurement date (n=4, *P*< 0.05, Tukey).

**Figure 2:**
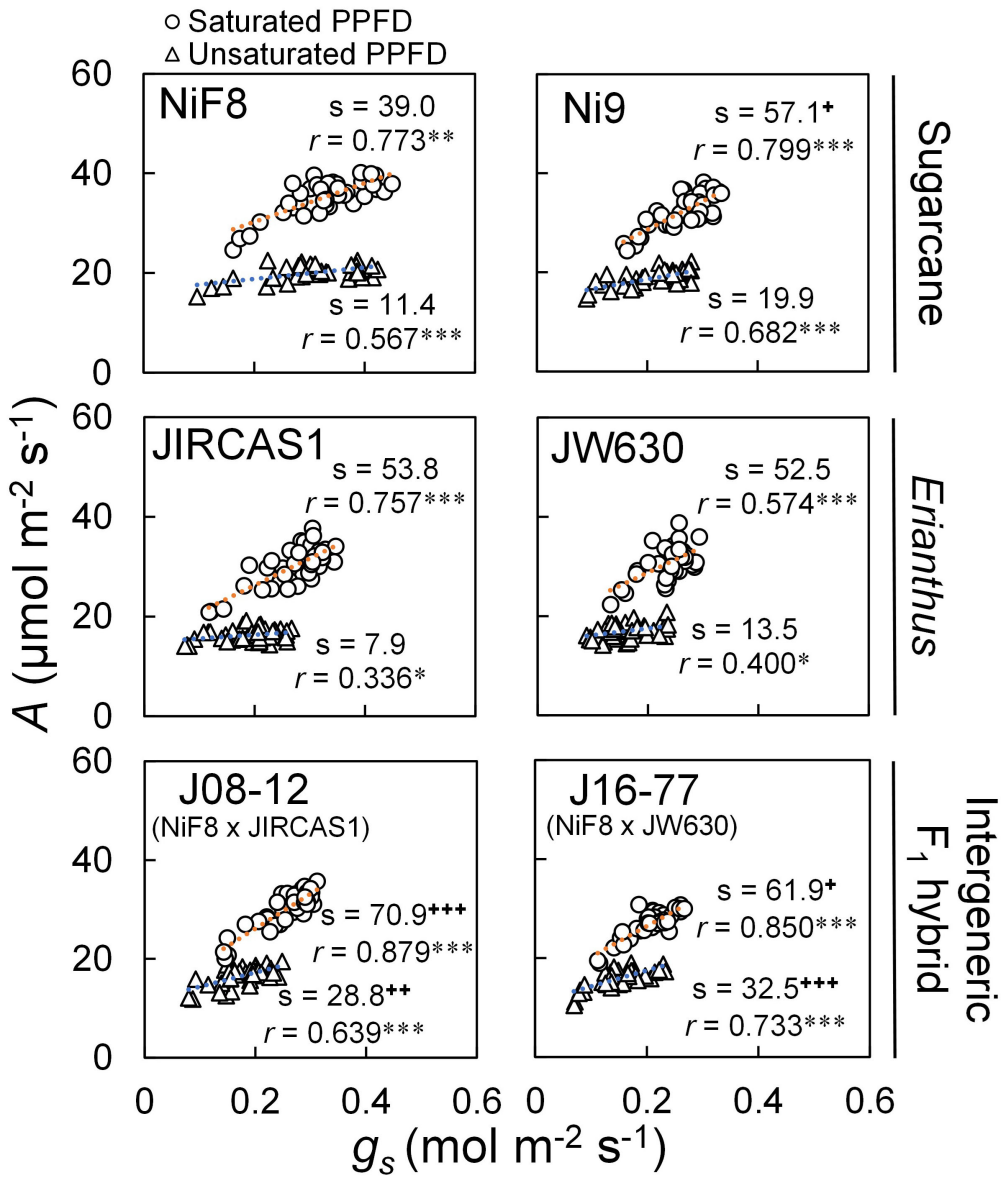
Phenotypic correlation between photosynthetic rate (*A*) and stomatal conductance (*g_s_*) under unsaturated and unsaturated light conditions. Gas exchange measurements were conducted at 2000 (○) and 500 (△) μmol m^-2^ s^-1^ of PPFD using LED for saturated and unsaturated light conditions, respectively. Orange and blue dotted line indicates linear regression line for measurements under saturated and unsaturated light conditions, respectively (n=40). “s” and “*r*” indicate regression slope and correlation coefficient, respectively. “*”, “**” and “***” indicate significant regression at *P* < 0.05, 0.01 and 0.001, respectively. “+”, “++” and “+++” indicate significantly different slope from one of NiF8 under each PPFD condition at *P* < 0.05, 0.01 and 0.001, respectively (*t*-test). Whereas intrinsic water use efficiency (*iWUE*) is expressed as the ratio of photosynthetic rate to stomatal conductance, the *A-g_s_* relation is a visual representation of the stomatal response of photosynthesis, whose regression equation slope indicates the ability of stomatal opening/closing to respond to soil moisture, allowing a linear interpretation of *iWUE*.

The relationship between *A* and *g_s_* was plotted for all measurements for both dry and wet treatments across each genotype (**Figure 2**). The correlation between *A* and *g_s_* was statistically significant under both unsaturated and saturated light conditions, with a steeper slope observed under saturated light than under unsaturated light. Among the genotypes, NiF8 exhibited a consistent tendency for higher *g_s_* (>0.3 mol m^-2^ s^-1^), regardless of light conditions. The slope under saturated light was significantly higher for Ni9, J08-12, and J16–77 than for NiF8, while JIRCAS1 and JW630 showed higher but not statistically significant trends. Under unsaturated light conditions, the slope was significantly higher for J08–12 and J16–77 compared with NiF8, whereas Ni9 and JW630 exhibited higher but non-significant trends.

**Figure 3:**
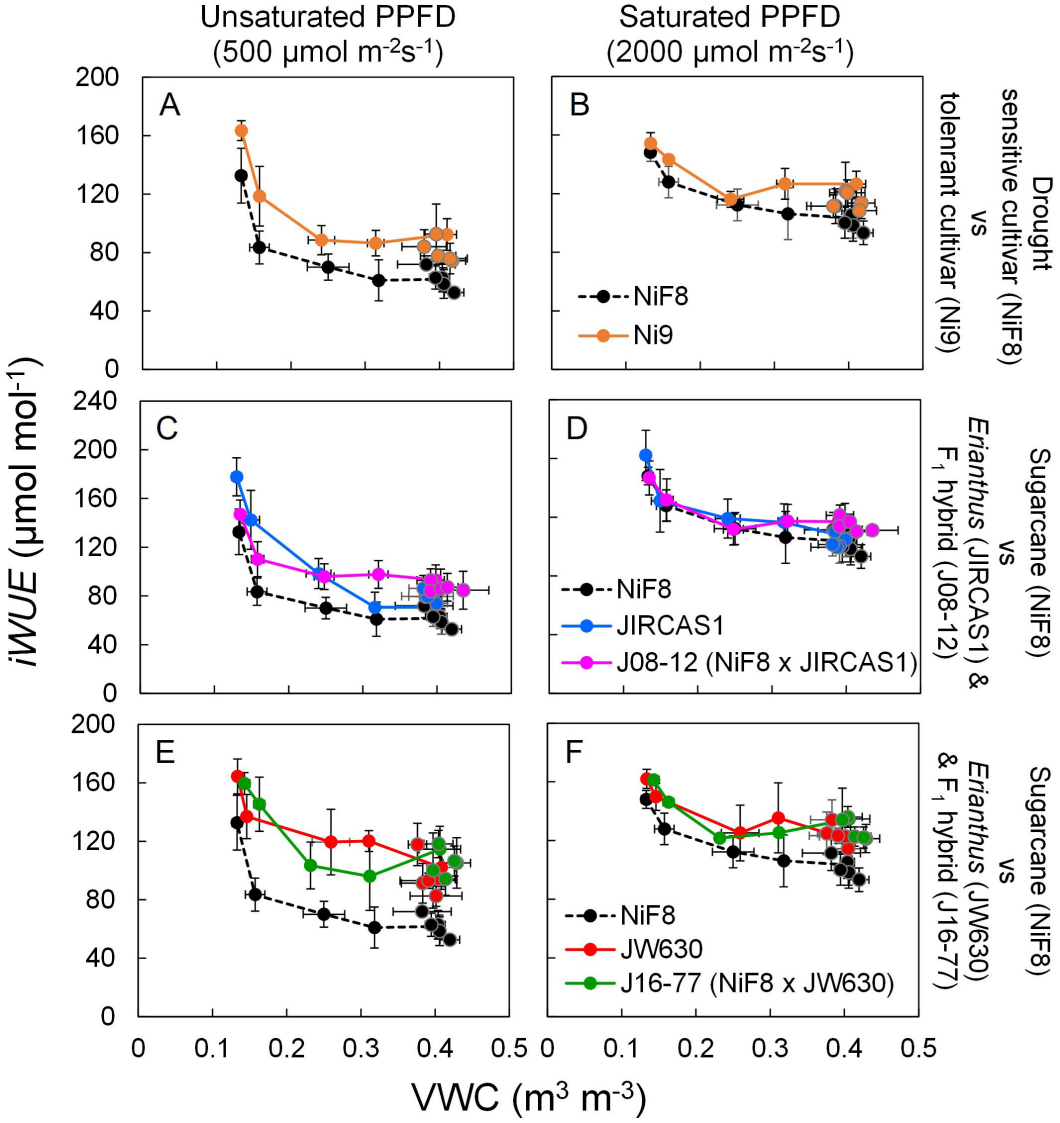
Responses of intrinsic water use efficiency (*iWUE*) to soil water changes under unsaturated and saturated light conditions. Average data under each soil water condition (wet or dry) at each measurement date were plotted with error bars for standard deviations (n=4 per genotype). Closed circle with and without line indicate the value under soil dry and wet conditions, respectively. Significant genotypic differences for *iWUE* were shown in the **Table 1** which shows the differences of values obtained at each date. **(A, B), (C, D), (E, F)** labels show the relations of NiF8 with Ni9, JIRCAS1, and JW630, respectively. **(A, C, E)** show data under unsaturated PPFD conditions while **(B, D, F)** show data under saturated PPFD condition.

The relation of *A*, *g_s_*, and *iWUE* to soil moisture was plotted to observe genotype-specific differences relative to NiF8 (**Supplementary Figures S5**, **S6**, **Figure 3**). Under saturated light conditions, NiF8 exhibited a higher *A* and more pronounced inter-genotype differences (**Supplementary Figure S5**). NiF8 also showed higher *g_s_*, with higher inter-genotype variation observed under unsaturated light than under saturated light conditions (**Supplementary Figure S6**). Both *A* and *g_s_* exhibited minimal inter-genotype differences under extremely dry conditions (VWC< 0.2 m^3^ m^-3^) (**Supplementary Figures S5**, **S6**). The differences in *iWUE* between genotypes were smaller under saturated light than under unsaturated light as well as under conditions of extreme dryness (VWC< 0.2 m^3^ m^-3^) compared to wetter conditions (**Figure 3**, **Table 1**). The *iWUE* of Ni9 consistently remained higher than that of NiF8 under both unsaturated and saturated light conditions, regardless of soil moisture levels (**Figures 3A, B**). Although the difference in *iWUE* between *Erianthus* JIRCAS1 and NiF8 was minimal under saturated light, the *iWUE* of JIRCAS1 tended to remain higher than that of NiF8 under unsaturated light conditions (**Figures 3C, D**). The *iWUE* of *Erianthus* JW630 was higher than that of NiF8 under both unsaturated and saturated light conditions (**Figures 3E, F**). The *iWUE* of the intergeneric hybrids J08–12 and J16–77 was comparable to or higher than that of their *Erianthus* parents JIRCAS1 and JW630, respectively (**Figures 3C–F**).

ANOVA results based on the mean of each genotype across all measurement conditions and dates indicated that gas exchange parameters were significantly influenced by soil moisture conditions, light conditions, and genotype (**Supplementary Table S1**). Among these parameters, *A*, *iWUE*, *C_i_*, *E*, and *A*/*E* (photosynthetic water use efficiency) were most strongly affected by PPFD, whereas *g_s_* was primarily influenced by genotype. Significant differences in *iWUE* between genotypes were observed on each measurement date, with JW630 and J16–77 typically exhibiting significantly higher *iWUE* than NiF8, except under saturated light conditions during the dry treatment (**Table 1**).

### 3.2 Comparison of leaf anatomical features among genotypes

The stomatal distribution of the test genotypes, including those of *Erianthus* and intergeneric hybrids, was amphistomatous, with a higher density of stomata on the abaxial surface than on the adaxial surface, consistent with other Poaceae species (**Supplementary Figure S7**, **Table 2**). ANOVA results revealed that genotype had a significant effect on all anatomical traits, whereas the effects of water regime and genotype–water interaction were relatively small (**Table 2**). Regardless of soil moisture conditions, NiF8 exhibited significantly higher stomatal density on the abaxial surface than JW630 and J16-77. The stomatal density on the adaxial surface was generally lower in NiF8 than in other genotypes, regardless of soil moisture conditions, with significant differences observed only in J08–12 under wet conditions and in JIRCAS1 and J16–77 under dry conditions. Overall, JW630 showed a lower stomatal density than the other genotypes. The ratio of adaxial to abaxial stomatal density under wet conditions was highest for JW630 and significantly higher for all other genotypes compared to NiF8. No significant differences in stomatal density ratios were observed between sugarcane cultivars or *Erianthus* accessions under dry conditions; however, *Erianthus* and intergeneric hybrids showed higher values compared to the two sugarcane cultivars. The interveinal distance did not differ among the sugarcane cultivars, whereas significant differences were found among *Erianthus* accessions (**Table 2**, **Supplementary Figure S8**). Specifically, *Erianthus* JW630 and the intergeneric hybrid J16–77 showed significantly longer interveinal distances than NiF8, whereas the intergeneric hybrid J08–12 showed a tendency for longer interveinal distance, although not significantly.

**Table 2:** Stomatal density and leaf interveinal distance of sugarcane, *Erianthus*, and intergeneric F_1_, hybrid under wet and dry conditions.

Treatment	Genotype		Stomatal density (no. mm^-2^)			Adaxial / Abaxial ratio	Interveinal distance (µm)
			Abaxial	Adaxial	Total		
Wet	Sugarcane	NiF8	177.9 c	92.8 a	270.7 b	0.52 a	122.7 ab
		Ni9	165.1 bc	101.2 ab	266.2 ab	0.61 b	121.1 ab
	*Erianthus*	JIRCAS1	160.7 bc	108.0 ab	268.6 ab	0.67 bcd	113.8 a
		JW630	134.6 a	98.4 ab	233.1 a	0.73 d	139.7 c
	F_1_ hybrid	J08-12 (NiF8 x JIRCAS1)	164.1 bc	114.9 b	276.1 b	0.68 cd	134.8 bc
		J16-77 (NiF8 x JW630)	147.8 ab	98.7 ab	246.4 ab	0.67 bc	151.8 c
Dry	Sugarcane	NiF8	177.0 c	93.7 ab	270.7 b	0.53 a	128.8 b
		Ni9	161.9 bc	89.8 a	251.7 ab	0.55 a	131.7 bc
	*Erianthus*	JIRCAS1	159.5 b	108.3 c	267.8 b	0.68 bc	111.8 a
		JW630	138.1 a	99.9 abc	238.0 a	0.72 c	147.0 cd
	F_1_ hybrid	J08-12 (NiF8 x JIRCAS1)	167.3 bc	106.1 bc	269.5 b	0.61 ab	135.9 bc
		J16-77 (NiF8 x JW630)	160.3 b	106.0 c	266.4 b	0.66 bc	153.5 d
*ANOVA* (%)		Genotype (G)	73.1 ***	43.7 ***	51.1 ***	80.7 ***	74.6 ***
		Water regime (W)	0.6	1.2	0.0	1.9 *	1.9
		G * W	3.1	15.4 *	9.2	4.1	2.0
		Residue	23.1	40.0	39.7	13.6	21.5

Different alphabet indicates significant difference between genotypes under each soil water (n=4, *P*< 0.05, Tukey). ANOVA was shown in the bottom column with percentage of each factorial variance to total variance. "*" and "***" indicate significance at *P*<0.05 and 0.001, respectively.

### 3.3 Comparison of biomass production response to drought among genotypes

ANOVA results indicated that both genotype and water regime significantly affected all parameters related to dry matter production (**Table 3**), except for NUE (**Supplementary Table S2**). Furthermore, the interaction between genotype and water regime was significant for all parameters, except for LAR and NUE. Genotypic differences in *gWUE* displayed varying trends across treatments. Under wet conditions, the genotypic differences in the *gWUE* of shoot dry mass were not significant, though the *gWUE* tended to be higher for the two sugarcane cultivars and the intergeneric hybrid J08–12 than for the two *Erianthus* accessions and the intergeneric hybrid J16-77. However, under dry conditions, genotypic differences in the *gWUE* of shoot dry mass were significant, with the two *Erianthus* accessions showing the lowest values, followed by the two sugarcane cultivars and the two intergeneric hybrids. Comparing the values between two treatments, the *gWUE* ratios of dry to wet conditions for average shoot dry mass tended to be higher for sugarcane and the intergeneric hybrids than for *Erianthus*, with similar trends observed for the *gWUE* of total dry mass. LAR was minimally affected by soil moisture conditions, with clear genotypic differences. The two *Erianthus* accessions exhibited significantly higher LAR values than the other genotypes. The shoot mass/root mass (S/R) ratios showed similar trends under both dry and wet conditions, being lower for *Erianthus* and higher for both sugarcane and intergeneric hybrids. The S/R ratio was the lowest for *Erianthus* JW630, and intermediate to higher for intergeneric hybrids compared to those for the parental genotypes. Under wet conditions, the stem elongation rate was significantly higher for the other genotypes than for *Erianthus* JW630, whereas under dry conditions, it was significantly higher or tended to be higher for the other genotypes than for the two *Erianthus* accessions. NUE variation among replicates was large, and genotypic differences were unclear (**Supplementary Table S2**).

**Table 3:** Shoot growth parameters of sugarcane, *Erianthus*, and intergeneric F_1_ hybrid under wet and dry conditions.

Treatment	Genotype		gWUE (gDW L^-1^)		LAR	Shoot / root ratio	Stem elongation rate
			Shoot	Total	(cm^2^ gDW^-1^)	(g g^-1^)	(cm day^-1^)
Wet	Sugarcane	NiF8	4.0 a	5.6 c	51.2 a	8.6 ab	2.0 b
		Ni9	4.5 a	4.9 abc	55.2 a	10.2 b	2.5 bc
	*Erianthus*	JIRCAS1	3.7 a	4.1 ab	74.9 b	8.2 ab	2.0 bc
		JW630	3.9 a	5.0 abc	74.1 b	4.8 a	1.0 a
	F_1_ hybrid	J08-12 (NiF8 x JIRCAS1)	5.0 a	5.4 bc	53.5 a	11.2 b	2.6 c
		J16-77 (NiF8 x JW630)	3.7 a	3.9 a	60.4 a	17.0 c	2.0 b
Dry	Sugarcane	NiF8	5.8 ab	8.7 b	47.1 a	7.7 c	1.4 bc
		Ni9	6.7 bc	7.7 ab	49.9 a	7.3 bc	1.8 c
	*Erianthus*	JIRCAS1	5.0 a	6.0 a	69.7 b	5.5 ab	1.1 ab
		JW630	5.3 ab	7.1 ab	66.4 b	4.8 a	0.8 a
	F_1_ hybrid	J08-12 (NiF8 x JIRCAS1)	7.4 c	8.8 b	48.4 a	6.2 abc	1.5 bc
		J16-77 (NiF8 x JW630)	7.7 c	8.4 b	54.7 a	11.5 d	1.6 c
Dry/Wet	Sugarcane	NiF8	1.46	1.55	0.92	0.89	0.69
		Ni9	1.50	1.57	0.90	0.71	0.70
	*Erianthus*	JIRCAS1	1.37	1.45	0.93	0.67	0.53
		JW630	1.36	1.41	0.90	1.01	0.82
	F_1_ hybrid	J08-12 (NiF8 x JIRCAS1)	1.50	1.62	0.91	0.56	0.57
		J16-77 (NiF8 x JW630)	2.07	2.13	0.91	0.68	0.79
	*ANOVA* (%)	Genotype (G)	20.0 ***	15.4 ***	76.6 ***	60.4 ***	47.6 ***
		Water regime (W)	56.3 ***	66.8 ***	6.8 ***	14.9 ***	32.0 ***
		G * W	9.1 **	5.7 *	0.3	7.2 *	7.3 **
		Residue	14.6	12.1	16.3	17.5	13.0

*gWUE* and LAR indicate gravimetric water use efficiency and leaf area ratio, respectively. Different alphabet indicates significant difference between genotypes under each soil water (n=4, *P*< 0.05, Tukey). ANOVA was shown in the bottom column with percentage of each factorial variance to total variance. "*", "**", and "***" indicate significance at *P*<0.05, 0.01, and 0.001, respectively.

**Figure 4:**
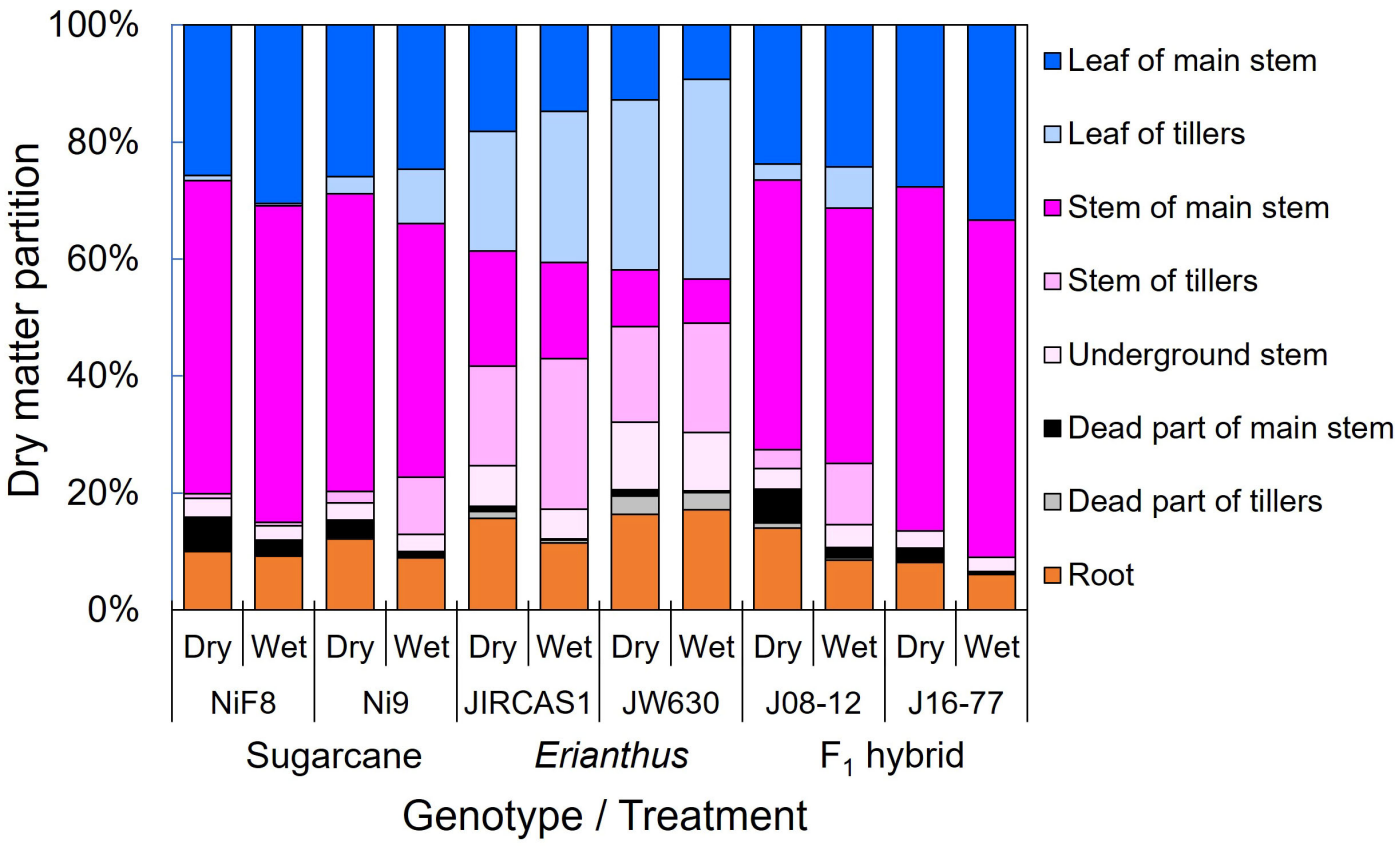
Dry matter partitions of sugarcane, *Erianthus*, and intergeneric F_1_ hybrid under wet and dry conditions. Blue (deep: main stem, light: tillers), purple (deep: main stem, semi-light: tillers, light: underground (stubble)), black (deep: main stem, light: tillers), and orange colors indicate leaf parts, stem parts, dead parts, and root parts, respectively. This figure visually shows the differences and similarities in dry matter distribution and supplements the dry matter distribution parameters expressed in terms of LAR and shoot/root ratio (**Table 3**). Here, we can visually see that sugarcane and intergeneric hybrids show similar dry matter partitioning.

Dry matter partitioning for each organ is shown in **Figure 4**. In sugarcane, a higher proportion of dry matter was allocated to the stem, with reduced allocation to the leaves due to drought, leading to an increase in dead tissue. In contrast, the *Erianthus* accessions exhibited higher partitioning to leaves and roots. Although intergeneric hybrids tended to increase root partitioning under drought, their overall dry matter allocation was similar to that of sugarcane, with more dry matter directed to the stems.

## 4 Discussion

### 4.1 Comparison of stomatal responses to soil water conditions in sugarcane and *Erianthus*

The relationship between *A* and *g_s_* (**Figure 2**) indicated that *Erianthus* exhibited a more sensitive stomatal response, with lower *g_s_* and lesser transpiration than the drought-susceptible cultivar NiF8, regardless of light conditions. This trend was particularly evident under unsaturated light conditions. In contrast, compared to that of the drought-tolerant cultivar Ni9, the *A vs g_s_* slope for *Erianthus* was not high, indicating that stomatal responsiveness in *Erianthus* was not necessarily higher than that in sugarcane (**Figure 2**). A key feature of gas exchange in *Erianthus*, as compared to sugarcane, is the high stomatal responsiveness while maintaining *g_s_* at consistent low levels, which indicates the presence of an underlying anatomical mechanism (Lawson and Blatt, 2014; Bertolino et al., 2019). Typically, longer interveinal distances and fewer stomata result in lower *g_s_* (Kawamitsu et al., 2002; Xu and Zhou, 2008; Pitaloka et al., 2022). When factors affecting stomatal responses, such as water status (soil moisture, VPD, etc.) and solar radiation, are variable, the stomatal reactivity—the ability to adjust stomatal opening and closing in response to these factors—plays a critical role in maintaining high *iWUE* (Kawamitsu et al., 1993; Tominaga et al., 2014; Matthews et al., 2017; Dinh et al., 2019; Lawson and Vialet-Chabrand, 2019; Ozeki et al., 2022). A better stomatal response has been reported in leaves that are more amphistomatous, with a higher distribution of stomata on the adaxial surface relative to the abaxial surface (Haworth et al., 2018; Drake et al., 2019; Xiong and Flexas, 2020). Amphistomatous leaves contribute to maintaining optimal leaf water status in response to transpiration demand. When stomata are open, the temperature gradient between the atmosphere, stomatal cavity, and leaf chloroplast is reduced (Drake et al., 2019), suggesting that stomatal responses to VPD—the driving force for transpiration—can be effectively regulated (Kawamitsu et al., 1993, 2002). Furthermore, in C_4_ grasses, more stomata on the adaxial surface can increase the surface area of mesophyll cells in contact with intracellular air space, enhancing *iWUE* and mesophyll conductance (Pathare et al., 2020). In the present study, the *Erianthus* species, particularly JW630, exhibited fewer stomata on the abaxial surface (resulting in a longer interveinal distance) and a more amphistomatous stomatal distribution (**Table 2**), which may explain its heightened stomatal responsiveness.

Furthermore, *Erianthus* JIRCAS1 exhibited a trend toward higher *iWUE* than the susceptible cultivar NiF8 under both wet and dry conditions, although its *iWUE* was not consistently higher than that of the drought-tolerant cultivar Ni9 (**Figure 3**, **Table 1**). In contrast, *Erianthus* JW630 consistently showed significantly higher *iWUE* than both NiF8 and Ni9. The high *iWUE* of *Erianthus* JW630 was likely attributed to leaf anatomy, including low stomatal density, (**Table 2**), leading to low *g_s_*, and may have a different physiological mechanism compared to that of drought-tolerant Ni9. The ability of *Erianthus* to maintain high *A* despite a low *g_s_* (that is, a high *iWUE*) may be linked to ultrastructural features such as mesophyll cell wall thickness and surface area in contact with the stomatal cavity, both of which are involved in bundle sheath leakiness (von Caemmerer and Furbank, 2003). Further investigation of gas exchange characteristics, such as *A*-*C_i_* curves, and anatomical features of this species will provide deeper insights into these mechanisms.

*Erianthus* exhibits genetically distinct lineages (Tsuruta et al., 2012, 2017, 2022) which influence variations in its morphological, ecological (Tagane et al., 2012), and agronomic (Terajima et al., 2022) traits. In the current study, variation in *iWUE* was observed between two *Erianthus* accessions (**Table 1**) which are classified into different genetic groups (Tsuruta et al., 2012, 2017). The accession JW630 was collected from Shizuoka Prefecture, a temperate region in Japan, while the origin of JIRCAS1 remains unknown. Previous studies have primarily focused on tropical accessions, such as the IJ series, which also exhibit high *iWUE* and related variations (Jackson et al., 2016; Li et al., 2017). These findings highlight the need for further investigation into the variation in leaf characteristics across different genotypic groups in *Erianthus*. Additionally, selecting *Erianthus* genotypes for improving drought tolerance in sugarcane will require considering both root system and aboveground traits.

### 4.2 Potential for enhancing sugarcane *gWUE* via *iWUE* improvement through intergeneric hybridization with *Erianthus*

Jackson et al. (2016) reported a strong correlation between *iWUE* and *gWUE* in sugarcane germplasm, with several *Erianthus* accessions showing higher values for both parameters compared to sugarcane. However, in the current study, high *iWUE* did not necessarily lead to high *gWUE* in *Erianthus*. This discrepancy may be attributed to differences in growing conditions: Jackson et al. (2016) conducted their trial in larger pots under outdoor conditions, while our study, was performed in smaller pots in a glasshouse. These differences may have limited branching in sugarcane varieties and caused greater root restriction in *Erianthus*. It is recommended that *gWUE* evaluation and screening in pot trials should take into account pot size and that evaluation of leaf traits at very early growth stages, rather than *gWUE*, would be more appropriate to validly evaluate genotypic differences under pot experiments. Despite this discrepancy with previous studies, low *iWUE* in sugarcane may result in low *gWUE* under field conditions, particularly under field canopy conditions, due to the larger leaf area and high LAR (or high LAI) of tillers. Additionally, because many leaves under a shaded canopy perform photosynthesis under low-light conditions (Almeida et al., 2022), where *iWUE*, which exhibits higher genotypic variation under low-light conditions, may have a more pronounced impact on *gWUE*. *Erianthus*, recognized for its drought-tolerance, may achieve high *gWUE* even at high LAR (caused by presence of many tillers), owing to its robust root system in the field (Terajima et al., 2023), in addition to its high *iWUE* (**Figure 3**, **Table 1**). The intergeneric F_1_ hybrids, which exhibited high *gWUE* under pot conditions**—**unlike the parental *Erianthus* accessions**—**showed high *iWUE* (**Table 1**) and demonstrated sugarcane-like dry matter partitioning characteristics (low LAR, high S/R ratio, and higher stem partitioning) (**Table 3**, **Figure 4**). Consequently, these hybrids may potentially maintain high *gWUE* even under field canopy conditions with high LAR. Although the intergeneric hybrids exhibited limited dry matter partitioning to roots in this study, which focused on relatively early growth under pot conditions, field studies have shown that the hybrid J08–12 forms roots with intermediate potential between parental species, exhibiting higher root mass and depth than sugarcane (Takaragawa et al., 2022; Terajima et al., 2023). These findings in the present study highlighted the "best of both worlds" scenario demonstrated by the hybrids, where they inherited high *iWUE* from *Erianthus* with favorable biomass partitioning characteristics from sugarcane. This fact could represent the ideal outcome for breeding drought-tolerant varieties via intergeneric hybridization with *Erianthus*. Further field trials will assess the relationships among *iWUE*, canopy coverage, root system formation, and *gWUE* using a hybrid population derived from several sets of parental genotypes.

The PPFD for gas exchange measurements had the greatest influence on *iWUE* (**Supplementary Table S1**). Genotypic differences in *iWUE* were particularly pronounced under unsaturated light (500 µmol m^-2^ s^-1^) conditions than under saturated light (2000 µmol m^-2^ s^-1^) conditions (**Figure 3**, **Table 1**). Additionally, the slope of the *A vs g_s_* curve was smaller, and the stomatal response was notably lower under unsaturated light conditions than under saturated light conditions (**Figure 2**). Therefore, *iWUE* screening under low-light conditions may prove effective and provide practical implications for developing high-throughput phenotyping protocols for drought tolerance screening. In contrast, such differences in stomatal responses due to varying light conditions suggest that obtaining stable results when measuring the response at multiple sites under field conditions may be challenging, especially in regions such as Okinawa (which comprise small islands and represent our study site), where the weather frequently shifts between cloudy and sunny within short time frames. Genotypic differences in *iWUE* vary depending on the measurement date; therefore, measuring within a moderate *g_s_* range (0.2–0.3 mol m^-2^ s^-1^, Jackson et al., 2016; 0.1–0.4 mol m^-2^ s^-1^, Natarajan et al., 2021) or averaging multiple measurements, is recommended (Jackson et al., 2016; Li et al., 2017). Considering this climate instability, investigating genotypic differences in response to fluctuating light conditions (Eyland et al., 2021; Tanaka et al., 2019) and exploring non-destructive methods for measuring daily variations in gas exchange, such as sap flow for transpiration (Contreras and Ozawa, 2005), are essential.

Gas exchange measurements are strongly influenced by environmental variations during data collection, which can compromise the stability and efficiency of the measurements. In recent years, the throughput of photosynthesis measurements has been enhanced by reducing measurement time through the use of closed-type equipment (Honda et al., 2021; Tanaka et al., 2021; Takaragawa and Matsuda, 2023). There have been no previous reports on high-throughput estimating and screening *iWUE* using UAVs and hyperspectral images, while component parameters for *iWUE* can be estimated by aerial image analysis: transpiration indices from leaf or canopy temperatures obtained from thermal images (Basnayake et al., 2016; Iseki and Olaleye, 2020; Natarajan et al., 2019) and photosynthetic activity using hyperspectral images (Kohzuma et al., 2021). However, despite recent advancements and attempts (Takaragawa et al., 2025), improvements in the measurement throughput of *iWUE*, which requires simultaneous measurement of photosynthesis and *g_s_*, remain incomplete.

Gas exchange is governed by complex biochemical processes influenced by metabolites, enzymes, and morphology (Sage et al., 2013). Among these, leaf morphological and anatomical traits, particularly stomatal characteristics, play a critical role in supporting gas exchange and mechanical function (Franks and Farquhar, 2007; Haworth et al., 2021). Although anatomical traits, such as stomatal density, are not sufficiently robust or universal enough to be used for species classification (Davis, 1987), they exhibit a smaller environmental variation compared to gas exchange characteristics and can show stable genotypic variation (Moreno-Sotomayor et al., 2002). The current study also demonstrated that environmental variation in leaf anatomical traits was relatively small as indicated by ANOVA results (**Table 2**, **Supplementary Table S1**). The leaf anatomical characteristics of the intergeneric F_1_ hybrids were intermediate between the parental genotypes, with intergeneric hybridization with *Erianthus* resulting in a progeny having longer interveinal distances, fewer stomata on the abaxial surface, and a higher stomatal distribution ratio (**Table 2**). These findings suggest that the improvement in *iWUE* through intergeneric hybridization was facilitated by changes in leaf anatomy. When examining hybrid populations, the throughput of morphological and anatomical observations may need to be enhanced through rapid image acquisition or other methods (Strock et al., 2022).

Although intergeneric hybridization offers potential for improving leaf traits of sugarcane, F_1_ hybrids typically exhibit lower sugar content with higher fiber content than sugarcane parents (Pachakkil et al., 2019), which discourages their direct utilization in breeding programs for sugar industry. Therefore, backcrossing using sugarcane variety must be performed to improve sugar content of hybrids, requiring a further investigation of leaf traits in the backcross populations.

## 5 Conclusions

**Figure 5:**
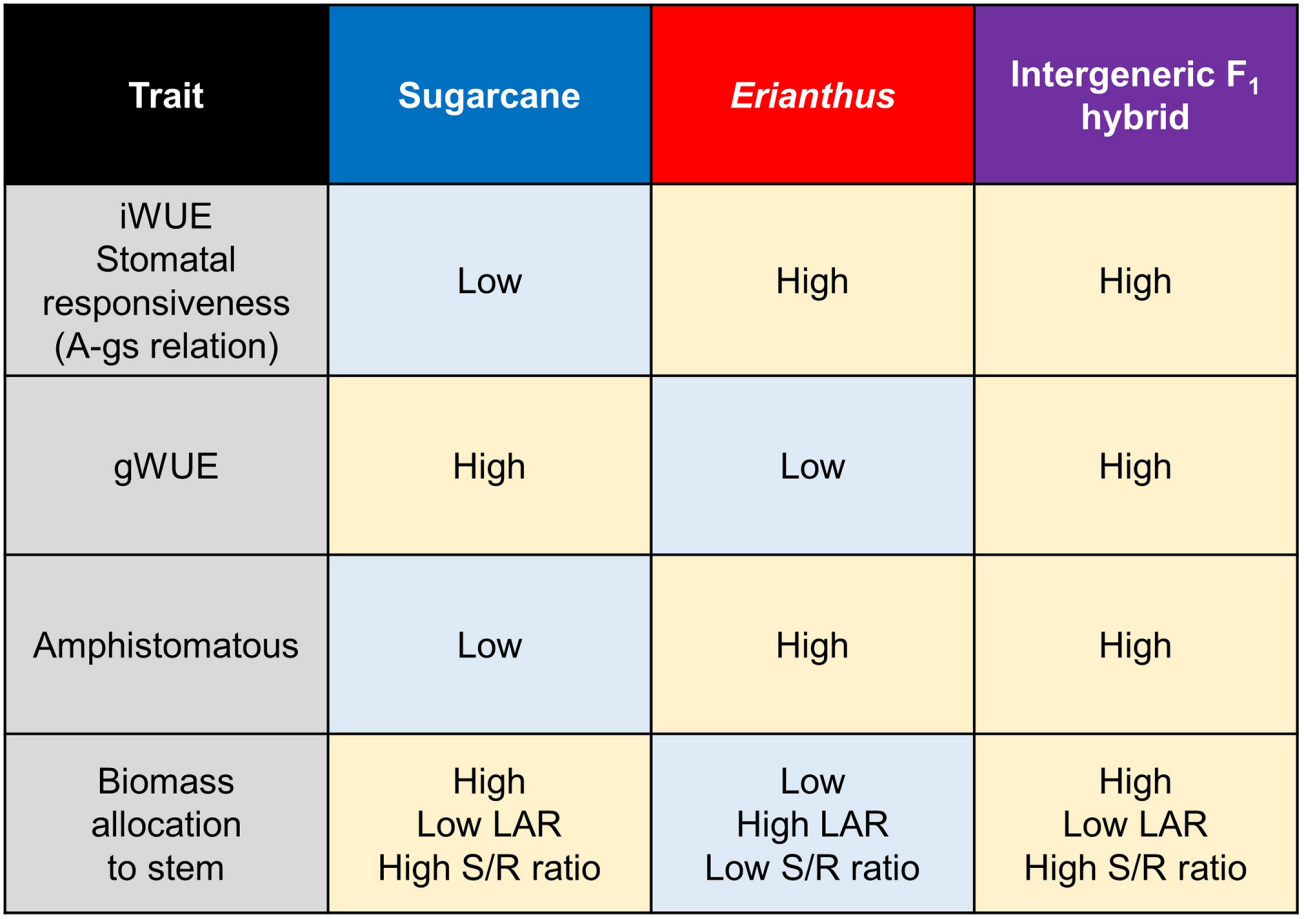
A schematic summary diagram for key differences between sugarcane, *Erianthus*, and intergeneric F_1_ hybrids. *iWUE*, *gWUE*, LAR, and S/R ratio indicate intrinsic water use efficiency, gravimetric water use efficiency, leaf area ratio, and shoot biomass/root baiomass ratio, respectively. Amphistomatous indicates high stomatal density ratio of adaxial to abaxial leaf surface. The diagram highlighted the "best of both worlds" scenario demonstrated by the hybrids.

We attempted to assess the potential for introducing the superior leaf traits of *Erianthus* into sugarcane by comparing the response of leaf traits to drought among sugarcane × *Erianthus* intergeneric F_1_ hybrids and their parental genotypes. In conclusion, the use of *Erianthus* germplasm, not drought-tolerant sugarcane cultivars, for improving drought tolerance in sugarcane remains a subject of debate. However, our study shows that incorporating *Erianthus* species into breeding programs could enhance the overall drought tolerance of sugarcane because intergeneric F_1_ hybrid exhibited favorable trait combinations inherited from both sugarcane and *Erianthus* parents (**Figure 5**). *Erianthus* has the potential to significantly improve not only leaf physiological and morphological characteristics, as demonstrated in the current study, but also the root system formation ability (Fukuhara et al., 2013; Takaragawa et al., 2022; Terajima et al., 2023). Future research will focus on comparing several F_1_ and BC hybrid populations, incorporating both drought-tolerant cultivars and *Erianthus*, to further assess their potential for improving drought resilience in sugarcane under field conditions.

## Data Availability

The raw data supporting the conclusions of this article will be made available by the authors, without undue reservation.
