# Supplementary material for: Improvement in the intrinsic water use efficiency of sugarcane by intergeneric hybridization with Erianthus arundinaceus
Source: Front Plant Sci. 2025 Nov 21;16:1649112. doi: 10.3389/fpls.2025.1649112 (PMC12678397; doi:10.3389/fpls.2025.1649112)

Table S1 ANOVA for gas exchange parameters

Note: Number in column indicates percentage of each factorial variance to total variance. *, **, and *** indicate significance at $P < 0.05$, 0.01, and 0.001, respectively.

Factor	A	g_s	$iWUE$	C_i	E	A/E
Genotype (G)	7.0 ***	33.6 ***	14.3 ***	7.8 ***	18.9 ***	8.6 ***
Water regime (W)	1.0 ***	10.7 ***	13.5 ***	8.9 ***	7.0 ***	9.6 ***
PPFD (P)	79.6 ***	17.3 ***	18.4 ***	42.3 ***	47.6 ***	20.8 ***
Date (D)	3.5 ***	10.6 ***	11.6 ***	6.6 ***	8.0 ***	5.8 ***
G * W	0.0	0.9 ***	0.7 *	0.6 **	0.2	0.4
G * P	0.7 ***	1.5 ***	1.7 ***	1.8 ***	0.4 **	2.7 ***
G * D	0.5 **	2.0 ***	2.0 **	1.6 **	1.4 ***	2.9 **
W * P	0.3 ***	0.0	0.7 ***	0.8 ***	0.0	2.3 ***
W * D	1.0 ***	9.8 ***	16.0 ***	10.7 ***	7.4 ***	12.4 ***
P * D	0.8 ***	0.2	1.8 ***	2.1 ***	0.2 *	3.8 ***
G * W * P	0.0	0.1	0.2	0.1	0.0	0.2
G * W * D	0.4 *	2.1 ***	1.0	0.8	0.8 *	1.3
G * P * D	0.3	0.6	1.1	0.9	0.4	1.8
W * P * D	0.2 ***	0.1	0.8 **	0.9 ***	0.0	2.5 ***
G * W * P * D	0.2	0.2	0.5	0.5	0.1	0.6
Residual	4.5	10.3	15.8	13.6	7.4	24.2

Table S2 Nitrogen use efficiency (NUE) of sugarcane, *Erianthus*, and intergeneric F_1 hybrid under wet and dry conditions.

Note: Different alphabet indicates significant difference between genotypes under each soil water (n=4, $P < 0.05$, Tukey). ANOVA was shown in the bottom column with percentage of each factorial variance to total variance.

Treatment		Genotype	NUE ($g\ gN^{-1}$)
Wet	Sugarcane	NiF8	183 a
		Ni9	166 a
	Erianthus	JIRCAS1	130 a
		JW630	176 a
	F_1 hybrid	J08-12 (NiF8 x JIRCAS1)	168 a
		J16-77 (NiF8 x JW630)	109 a
Dry	Sugarcane	NiF8	154 a
		Ni9	167 a
	Erianthus	JIRCAS1	139 a
		JW630	179 a
	F_1 hybrid	J08-12 (NiF8 x JIRCAS1)	174 a
		J16-77 (NiF8 x JW630)	116 a
Dry /Wet	Sugarcane	NiF8	0.84
		Ni9	1.01
	Erianthus	JIRCAS1	1.06
		JW630	1.02
	F_1 hybrid	J08-12 (NiF8 x JIRCAS1)	1.04
		J16-77 (NiF8 x JW630)	1.07
	ANOVA (%)	Genotype (G)	27.0 *
		Water regime (W)	0.0
		G * W	2.0
		Residue	70.9

Fig. S1 Confirmation of F_1 hybrid by PCR assay and flowcytometry

Note: Identification test for intergeneric hybridization was performed using PCR-based assay with 5S ribosomal DNA (5S rDNA) primers (D'Hont et al. 1995). Nuclear DNA content, shown in bottom table, was estimated using flow cytometry assay as another identification test (Pachakkil et al. 2019).

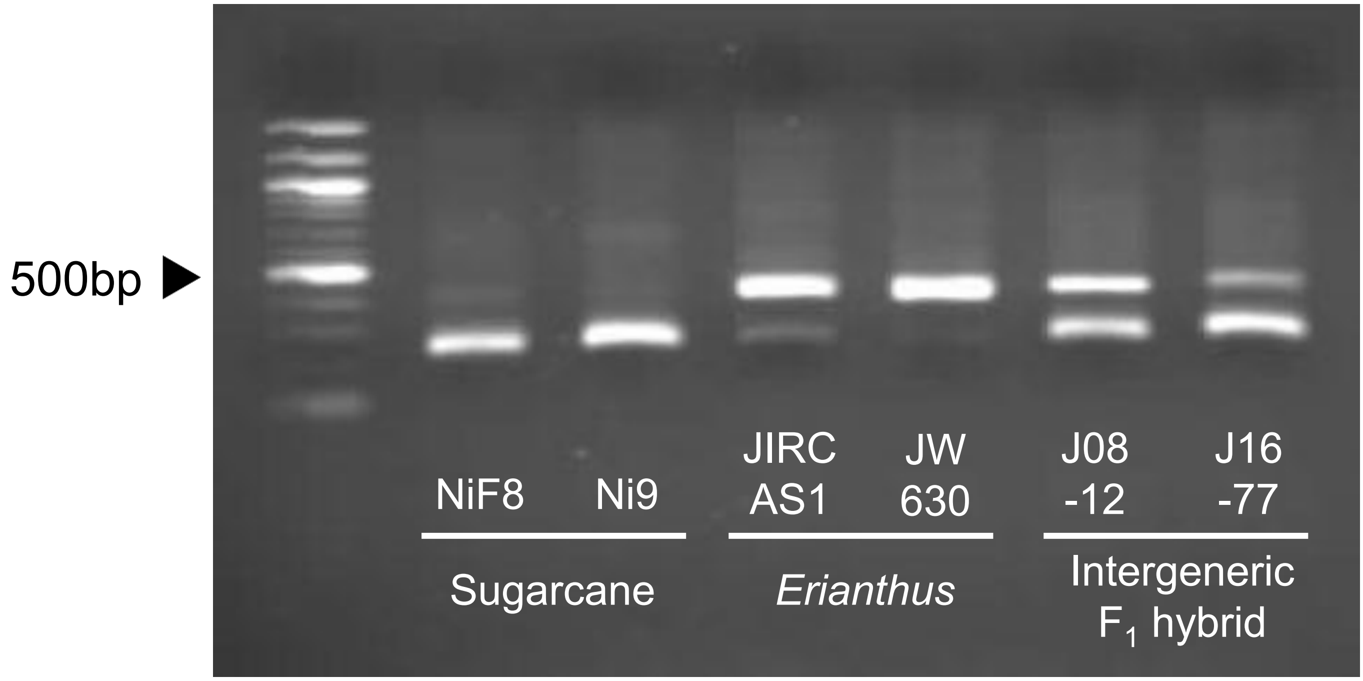

DNA content (pg/2C)	10.4	10.2	6.8	7.1	9.1	8.0

Fig. S2 Climatic conditions in the growth chamber.

Note: Orange solid and dotted line indicate mean air temperatures at daytime (6:00 – 18:00) and night (18:00 – 6:00), respectively. Blue solid and dotted line indicate mean relative humidity (RH) at daytime (6:00 – 18:00) and night (18:00 – 6:00), respectively. Purple solid line indicates daily cumulative radiation.

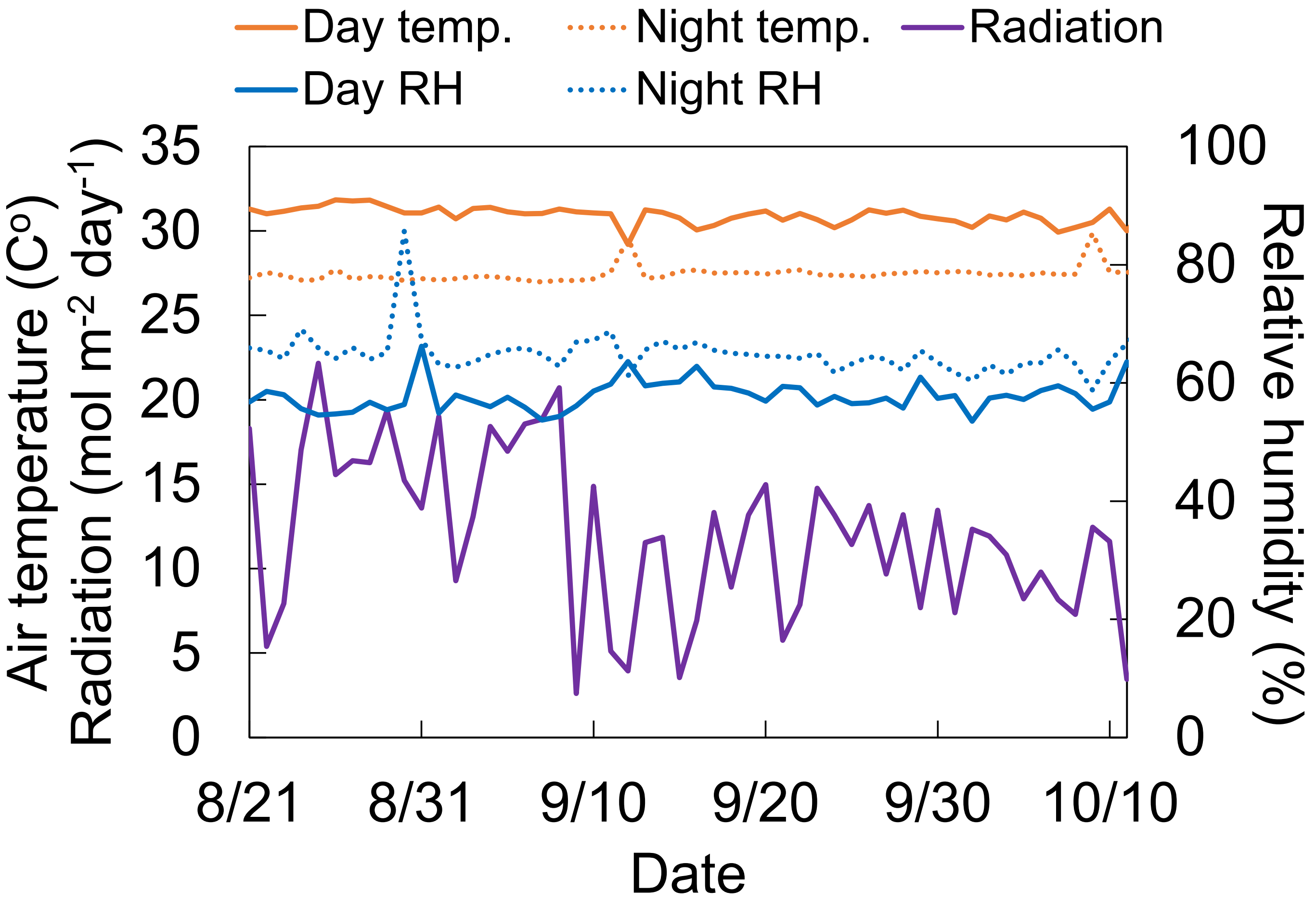

Fig. S3 Relation between soil matrix potential (pF) and volume water content (VWC).

Note: Circles indicate observed values (obs). pF values were obtained by conventional methods (pressure plate, gravity drainage column, and psychrometer). VWC values were obtained by gravimetric methods based on soil bulk density. To gain relation between pF and VWC, fitting of these values was performed according to van Genuchten (1980) as shown by an orange curve (est). Small table in the figure shows estimated VWC values at some important pF values.

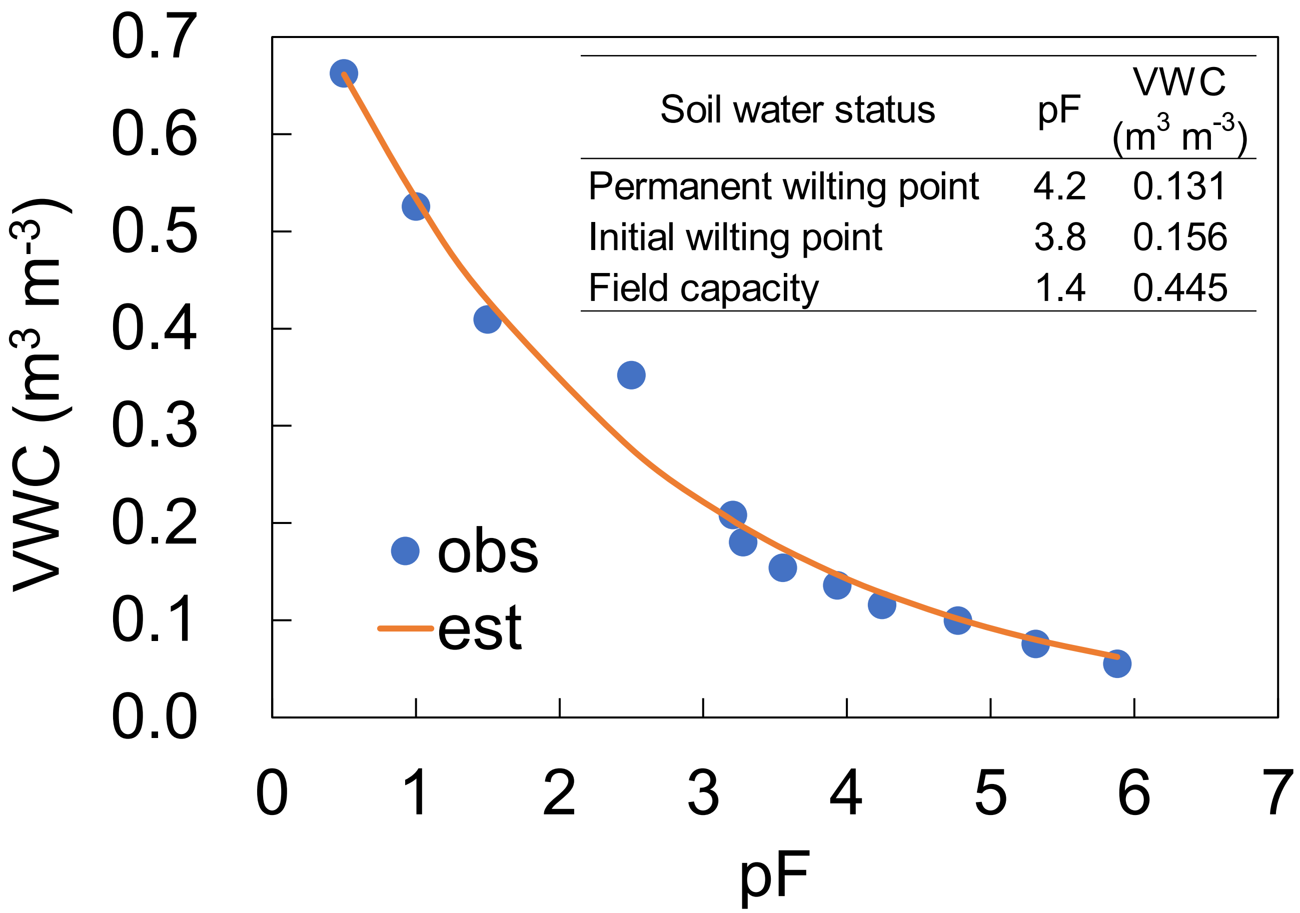

Soil water status	pF	VWC (m^3 m^{-3})
Permanent wilting point	4.2	0.131
Initial wilting point	3.8	0.156
Field capacity	1.4	0.445

Fig. S4 Changes of soil matrix potential (pF) at 15-cm depth under wet and dry conditions.

Note: Blue and red lines indicate pF values under wet and dry conditions, respectively. Manual irrigation control and stress treatment were started from 8/20 and 8/30, respectively. Arrows indicate five dates for gas exchange measurement. pF values were estimated by the relation between pF and volume water content as shown in Fig. S2

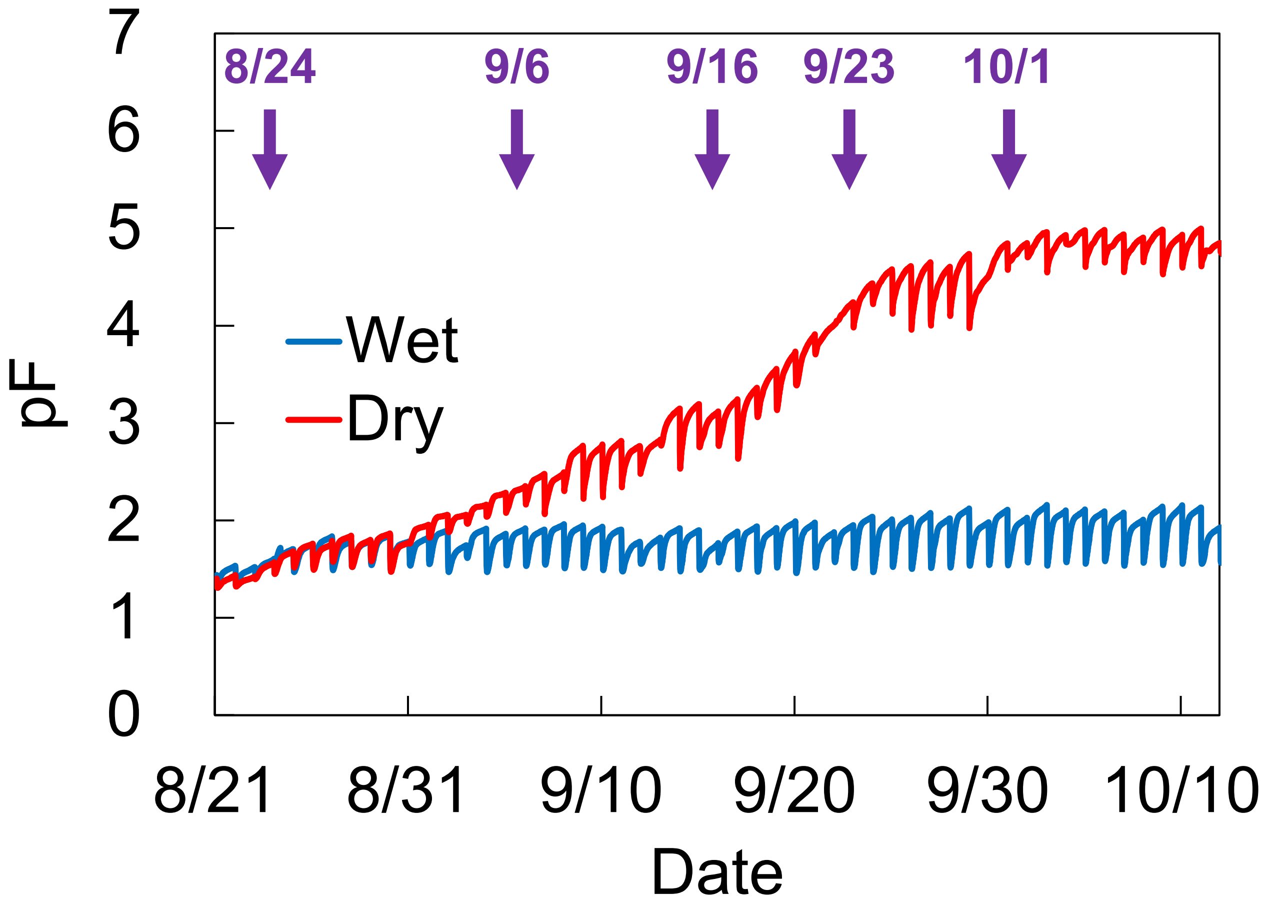

Fig. S5 Responses of Photosynthetic rate (*A*) to soil water changes under unsaturated and saturated light conditions.

Note: Average data under each soil water condition (wet or dry) at each measurement date were plotted (n=10 per genotype). Closed circle with and without line indicate the value under soil dry and wet conditions, respectively.

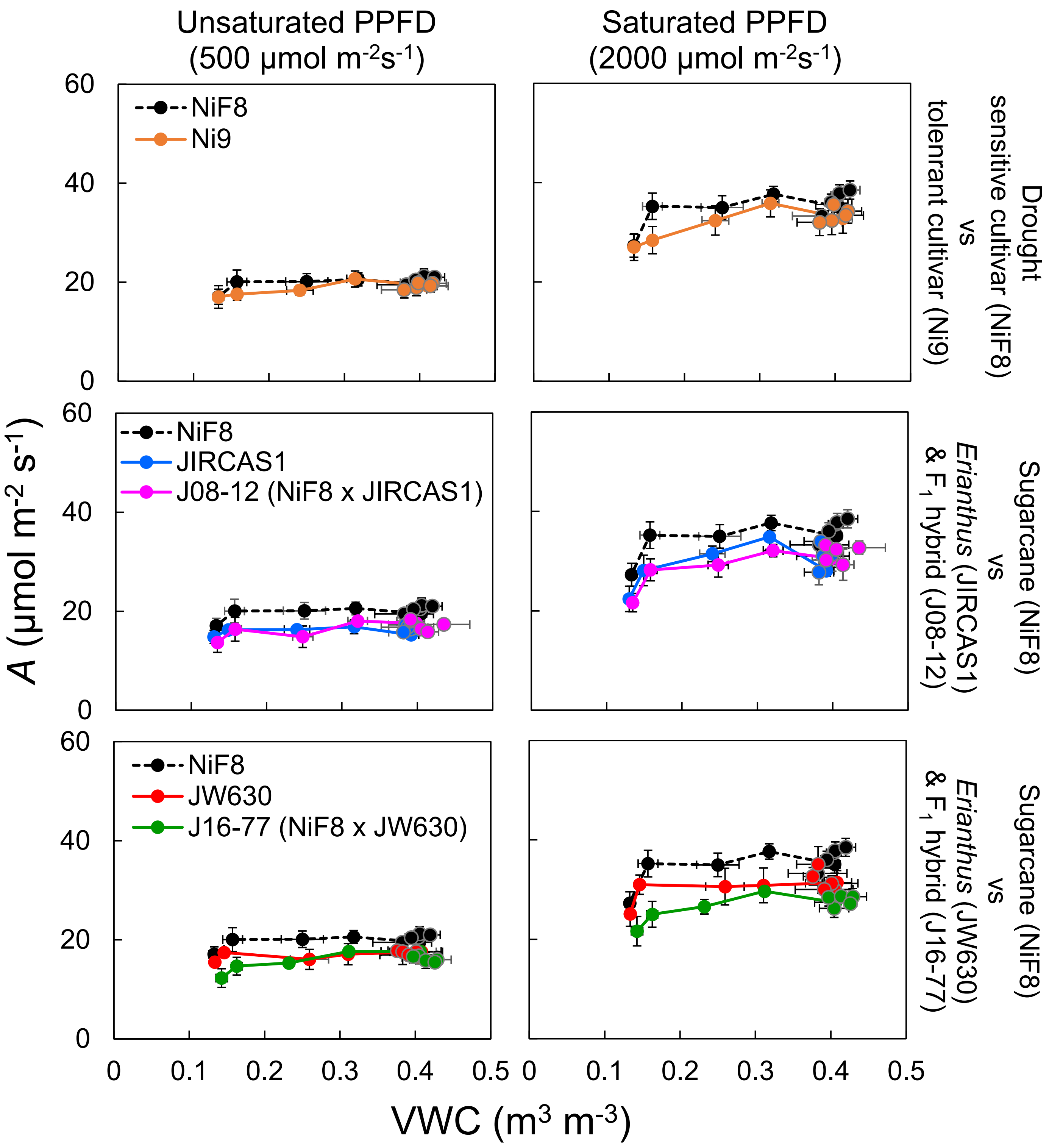

Fig. S6 Responses of stomatal conductance (g_s) to soil water changes under unsaturated and saturated light conditions.

Note: Average data under each soil water condition (wet or dry) at each measurement date were plotted (n=10 per genotype). Closed circle with and without line indicate the value under soil dry and wet conditions, respectively.

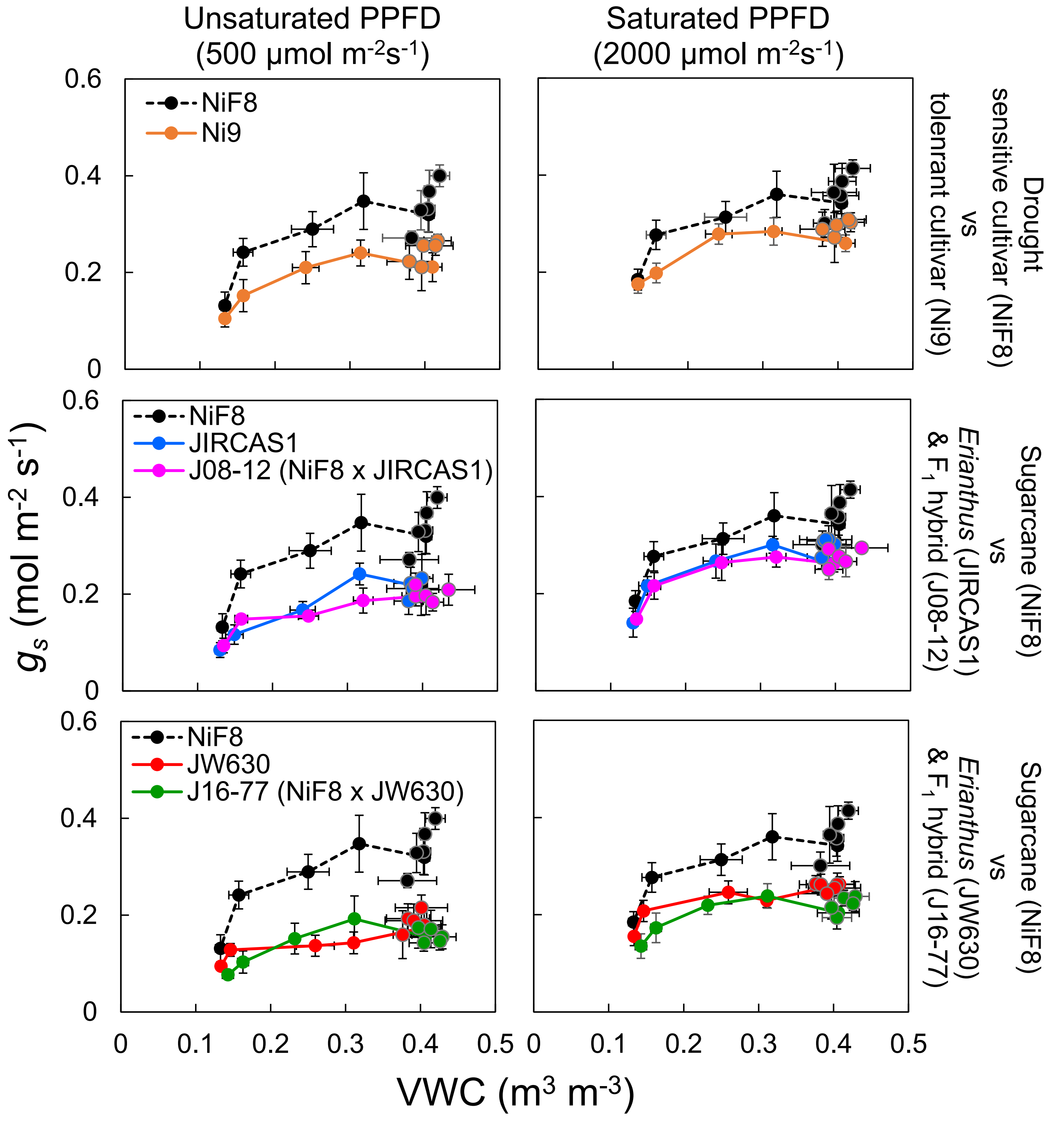

Fig. S7 Stomatal replica of genotypes examined in the present study

Note: Bar scale indicates 0.2 mm. Stomata replicas of leaf samples under wet conditions were shown

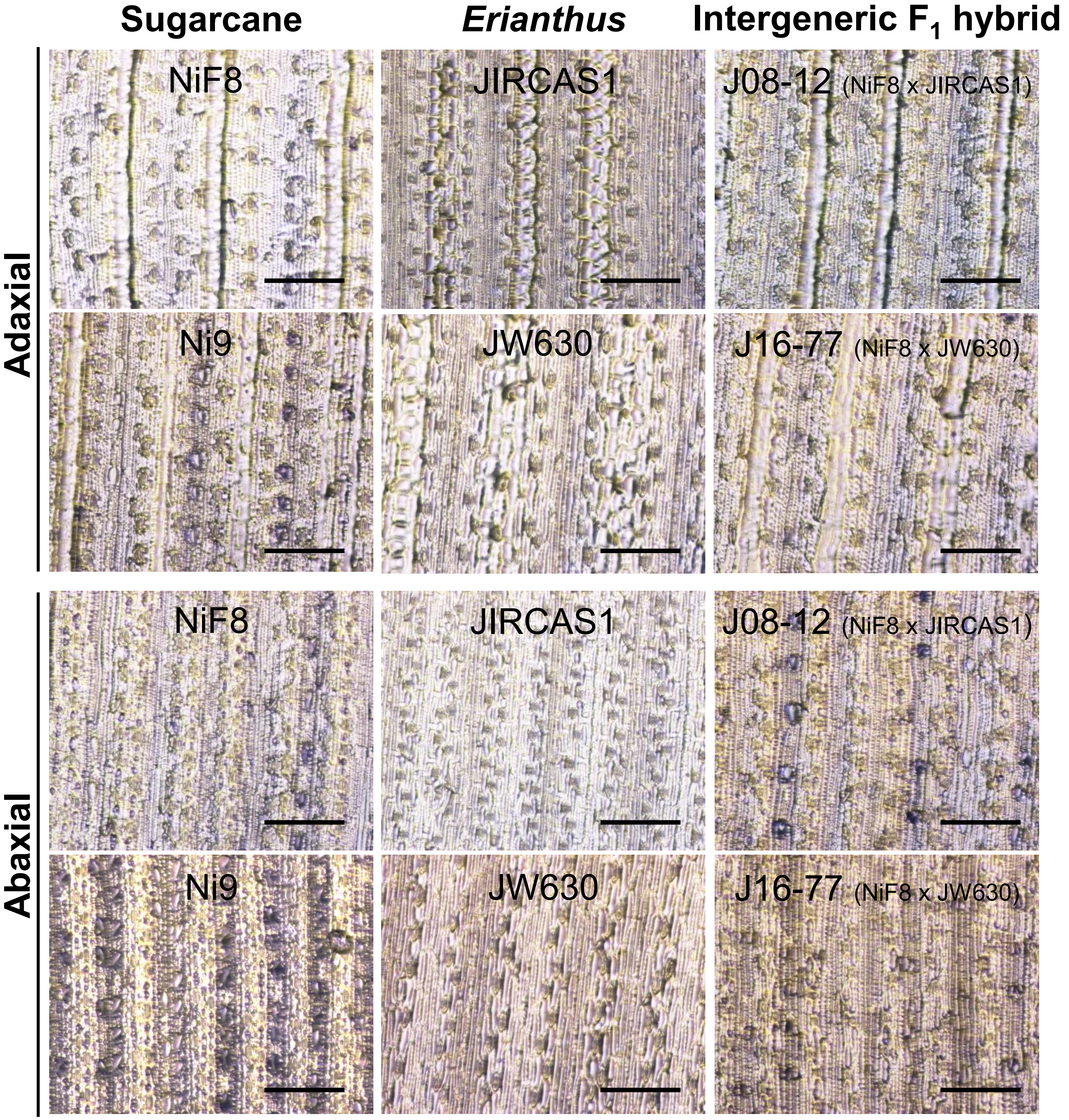

Fig. S8 Transverse leaf section of genotypes examined in the present study

Note: Bar scale indicates 0.2 mm. Sections of leaf samples under wet conditions were shown. Upper side of photo is an adaxial side of leaf.

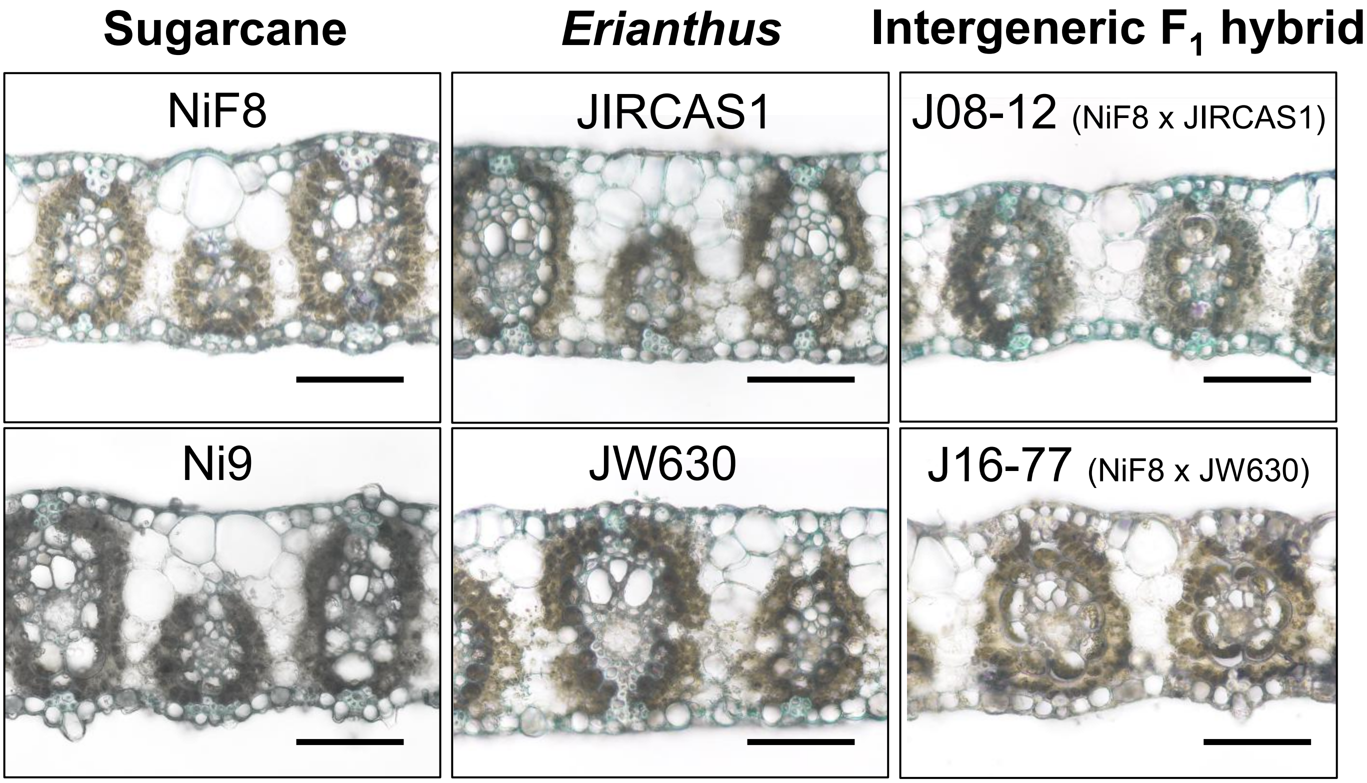

Supplement: Supplementary file 1 [file DataSheet1.pdf]
